# Coronary Slow Flow Phenomenon and Atrioventricular Block: A Case Report

**Published:** 2017-04

**Authors:** Mohammad Masoumi, Khadije Mohammadi

**Affiliations:** 1 *Physiology Research Center, Institute of Neuropharmacology, Kerman University of Medical Sciences, Kerman, Iran.*; 2 *Shafa Hospital, Kerman University of Medical Sciences, Kerman, Iran.*

**Keywords:** *Atrioventricular block*, *Coronary angiography*, *Coronary vessels*

## Abstract

The coronary slow flow phenomenon (CSFP) is characterized by a delayed coronary blood flow in the absence of an obstructive coronary artery disease. Although the relation between the CSFP and myocardial ischemia has been reported previously, there is no knowledge about the relationship between the CSFP and the conduction system disorder. In this case report, we describe a patient with the CSFP presenting with complete heart block (CHB). The patient was a middle-aged woman with a history of diabetes, hypertension, and prior Coronary Care Unit admission presenting with dizziness, lightheadedness, and presyncope. Electrocardiography revealed CHB with no significant ST-T change. Cardiac enzymes and other routine lab tests were normal. The patient underwent temporary pacemaker implantation. Due to persistent atrioventricular block and suspicion of ischemic heart disease, she underwent coronary angiography, which showed the CSFP and no significant stenosis. The patient was discharged after permanent pacemaker implantation and remained asymptomatic at 3 months' follow-up.

## Introduction

The coronary slow flow phenomenon (CSFP) is a disease characterized by the slow passage of the angiographic contrast in the coronary arteries in the absence of stenosis in the epicardial vessels. The overall incidence of the CSFP is 1% among patients undergoing coronary angiography and 4% among those presenting with the acute coronary syndrome.^1^ The CSFP has various presentations: from mild chest discomfort to ST-segment elevation myocardial infarction. It can, therefore, have severe morbidity and mortality implications and significantly hamper the quality of the life of those affected.^2^ In this paper, we describe a patient with the CSFP who developed a third-degree atrioventricular (AV) block.

## Case Report

**Figure 1:**
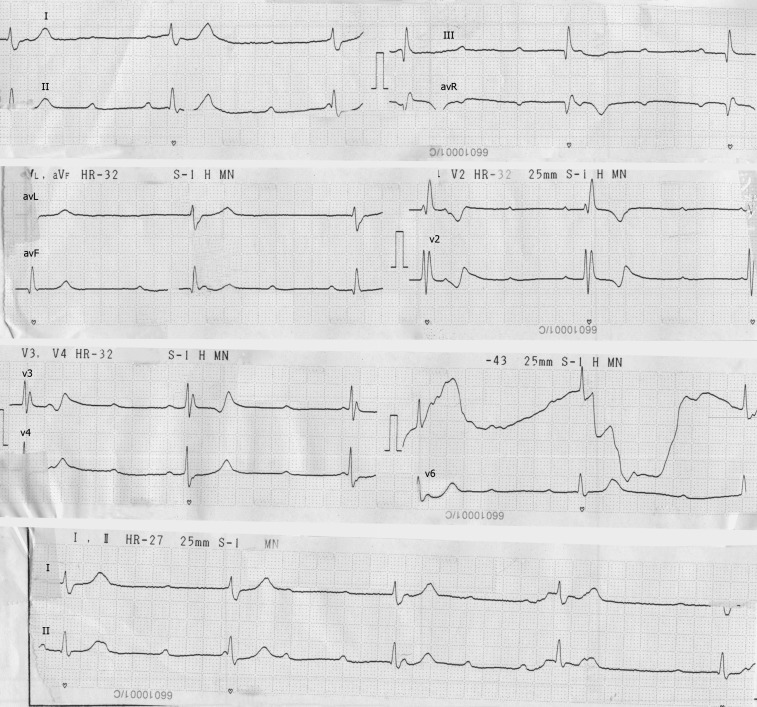
Electrocardiogram of the patient showing complete heart block with no significant ST-T change

A 57-year-old woman with a previous history of diabetes, hypertension, and Coronary Care Unit admission (due to chest pain and dyspnea on exertion) referred to our hospital with lightheadedness, dizziness, and presyncope. The patient's drug history revealed that she was taking Aspirin (ASA), Atorvastatin, and Losartan. Upon presentation, her heart rate was 30 beats per minutes and electrocardiography revealed complete heart block (CHB) with no significant ST-T change (Figure 1). Cardiac enzymes (troponin and Creatine phosphokinase-MB [CPK-MB]) were negative in serial sampling.

Laboratory findings were within normal limits. Transthoracic echocardiography illustrated normal left ventricular size, ejection fraction of 60%, diastolic dysfunction grade 1, and no valvular disorders.

**Figure 2:**
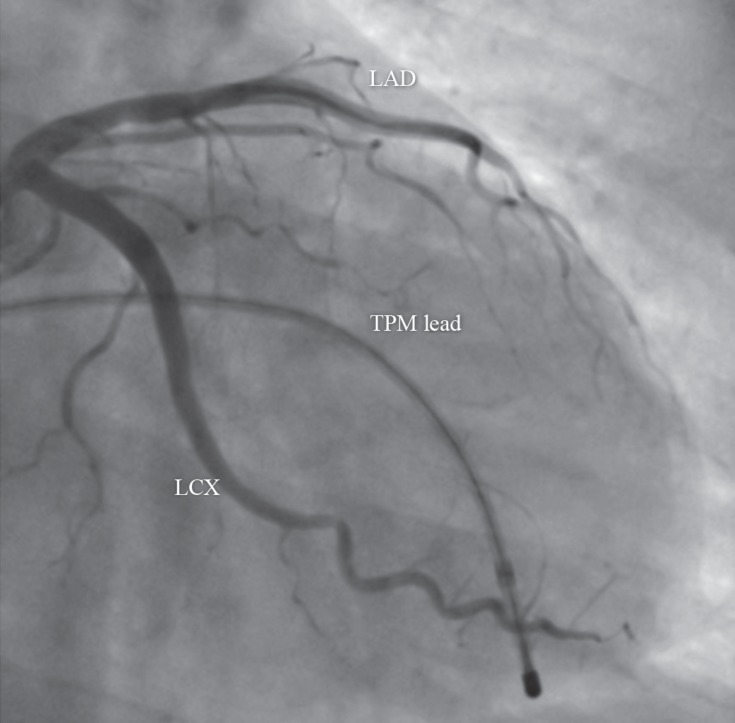
Right anterior oblique coronary angiographic view, showing no significant stenosis in the epicardial coronary arteries

LAD, Left anterior descending artery; LCX, Left circumflex artery; TPM, Temporary pacemaker

Given the patient's symptomatic third-degree AV block, she underwent temporary pacemaker implantation. However, in spite of anti-ischemic therapy, her CHB persisted and she underwent coronary angiography, which demonstrated normal coronary arteries without evidence of coronary vasospasm or myocardial bridge (Figure 2). Nevertheless, there was a slow flow in the left anterior descending artery (LAD) and right coronary artery (RCA). Corrected Thrombolysis in Myocardial Infarction (TIMI) frame count of the LAD was 34 (the Gibson method with a correction factor of 1.7 for the LAD), and the TIMI frame count of the RCA was 28.

As there was no significant stenosis, the patient was discharged after the implantation of a permanent pacemaker. The patient’s complaints improved after the implantation of the pacemaker and she remained asymptomatic during her 3 months’ follow-up.

## Discussion

The CSFP was first described by Tambe et al.^3^ in 1972, and is defined as delayed opacification of the coronary vessels during angiography without any evidence of an obstructive disease. Quantitatively, it is measured as an increased TIMI frame count. The TIMI frame count, introduced by Gibson, is a reproducible index of the coronary flow and represents the number of cine frames required for the contrast to reach a prespecified distal coronary artery landmark.^4^

The various presentation of the CSFP ranges from stable or unstable angina, non-ST-elevation myocardial infarction, ST-elevation myocardial infarction, nonsustained ventricular tachycardia, and chest discomfort.^2^ Yilmaz et al.^5^ reported higher total cholesterol and low-density lipoprotein cholesterol levels among their patients with the CSFP. The authors also stated that the body mass index was higher and the metabolic syndrome was more frequent in their CSFP subjects than in their control group. In a case-series, Azzarelli et al.^6^ reported that their CSFP patients often presented with recurrent chest pain.

Our patient presented with dizziness and presyncope due to the AV block, which was ultimately diagnosed as the CSFP.

Many conditions such as the fibrosis and sclerosis of the conduction system, ischemic heart diseases, and increased vagal tone as well as drugs such as Digitalis, calcium antagonists, beta blockers, Quinidine, and Amiodarone can cause a third-degree AV block. Our patient was not on any medication that would have caused the AV block. Her medical history contained no conditions such as infectious and metabolic diseases, rheumatic or degenerative diseases, neuromuscular disorders, or infiltrative processes that could have affected the AV node. Accordingly, we suggest that this AV block might have been due to the CSFP. The slow flow in the RCA might have caused an ischemia in the AV nodal artery, progressing to an AV block. To our knowledge, a causal relationship between the CSFP and the AV block has not been reported previously. The only exception is a study by Acikel et al.^7^, who reported an association between intermittent left bundle branch block and the CSFP in a patient presenting with the acute coronary syndrome.

## Conclusion

The case presented herein demonstrates that the atrioventricular conduction block can be a presentation of the coronary slow flow phenomenon and that it can have a diverse spectrum of presentations.

## Notes:

***This paper should be cited as:*** Coronary Slow Flow Phenomenon and Atrioventricular Block: A Case Report. J Teh Univ Heart Ctr 2017;12(2):85-87.
